# Supplementary material for: Mice with hyper-long telomeres show less metabolic aging and longer lifespans
Source: Nat Commun. 2019 Oct 17;10:4723. doi: 10.1038/s41467-019-12664-x (PMC6797762; doi:10.1038/s41467-019-12664-x)
Supplement: Supplementary file 1 — Supplementary Information [file 41467_2019_12664_MOESM1_ESM.pdf]

Supplementary Information

Mice with hyper-long telomeres show less metabolic aging and longer lifespans

Muñoz-Lorente *et al.*

Supplementary figure 1

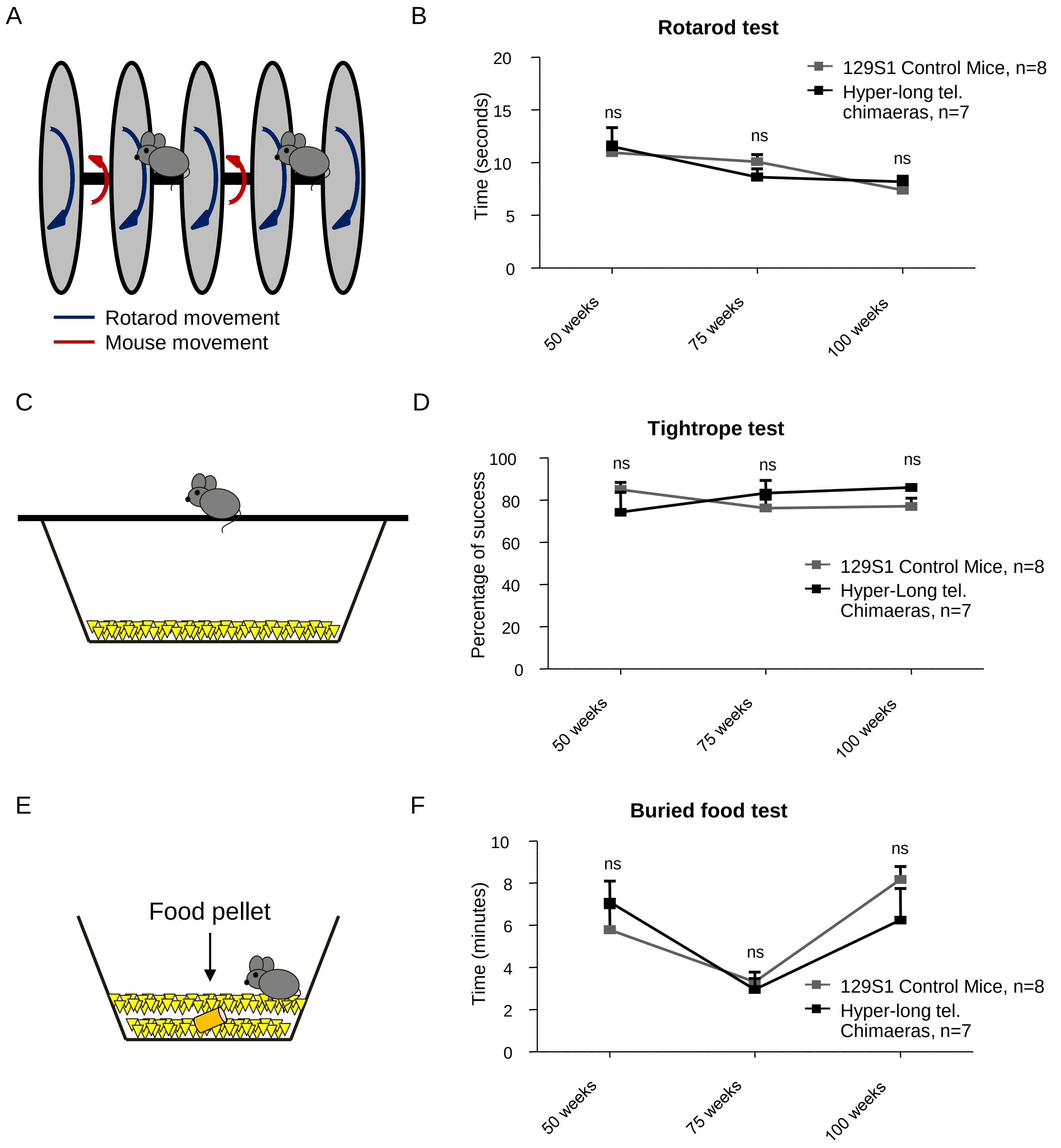

Supplementary Figure 1. Mice with hyper-long telomeres have normal cognitive capabilities. A-F. Different cognitive assays were performed in hyper-long telomere mice and age-matched controls at 50, 75 and 100 weeks of age. In order to evaluate neuromuscular endurance we performed the rotarod test (A-B) which measures the time that mice are able to stay on a rotating platform with accelerated movement without falling, for coordination we performed tightrope test which evaluates the capability of the mice to stay on a rope without falling during at least 1 minute (C-D) and for sensory perception we performed the buried food test (E-F) in which we measured the ability of mice to find a buried food pellet after 16h fasting. Error bars represent standard error. *t*-test was used for statistical analysis. The number of mice is indicated in each case.

Supplementary figure 2

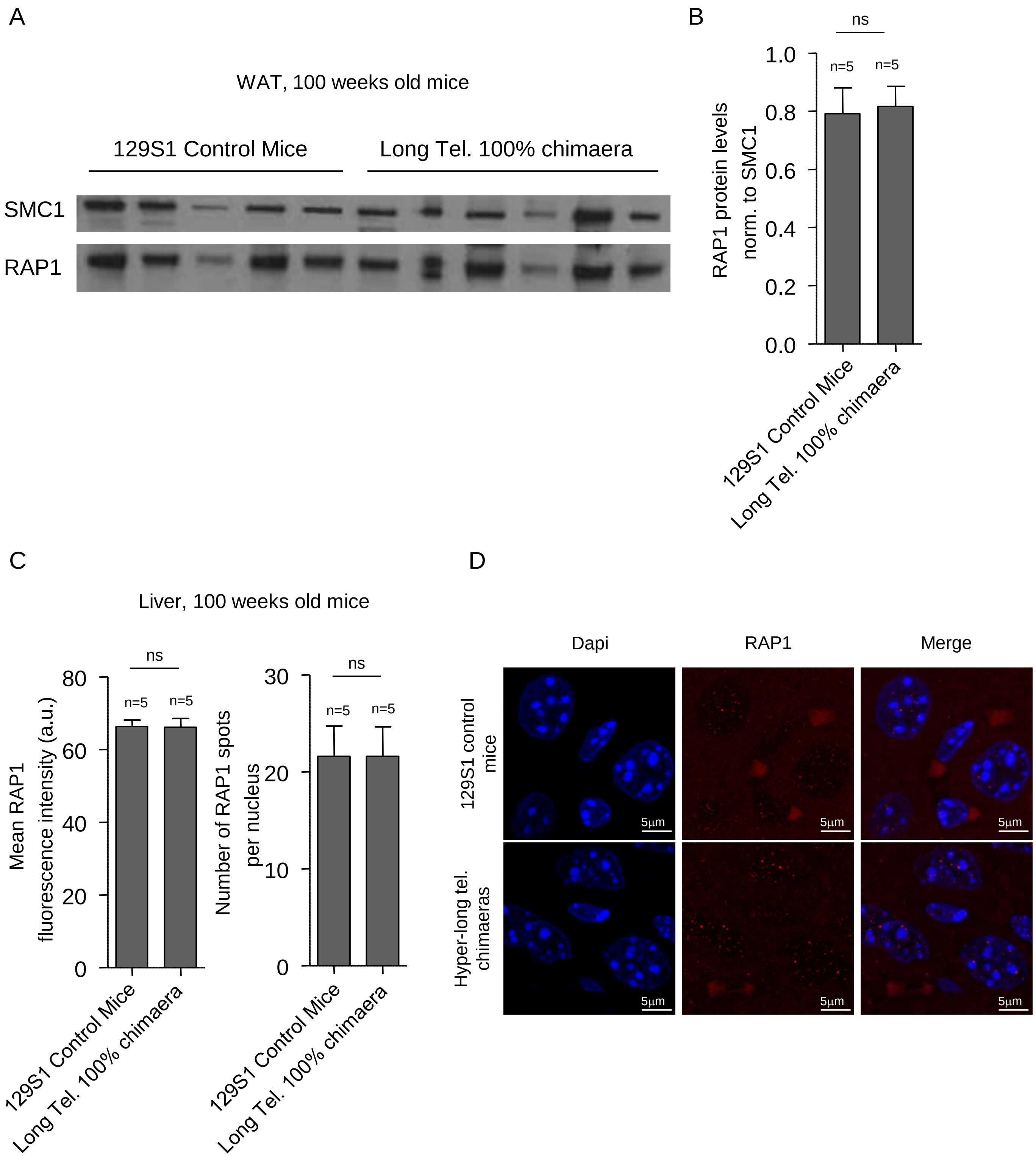

Supplementary figure 2

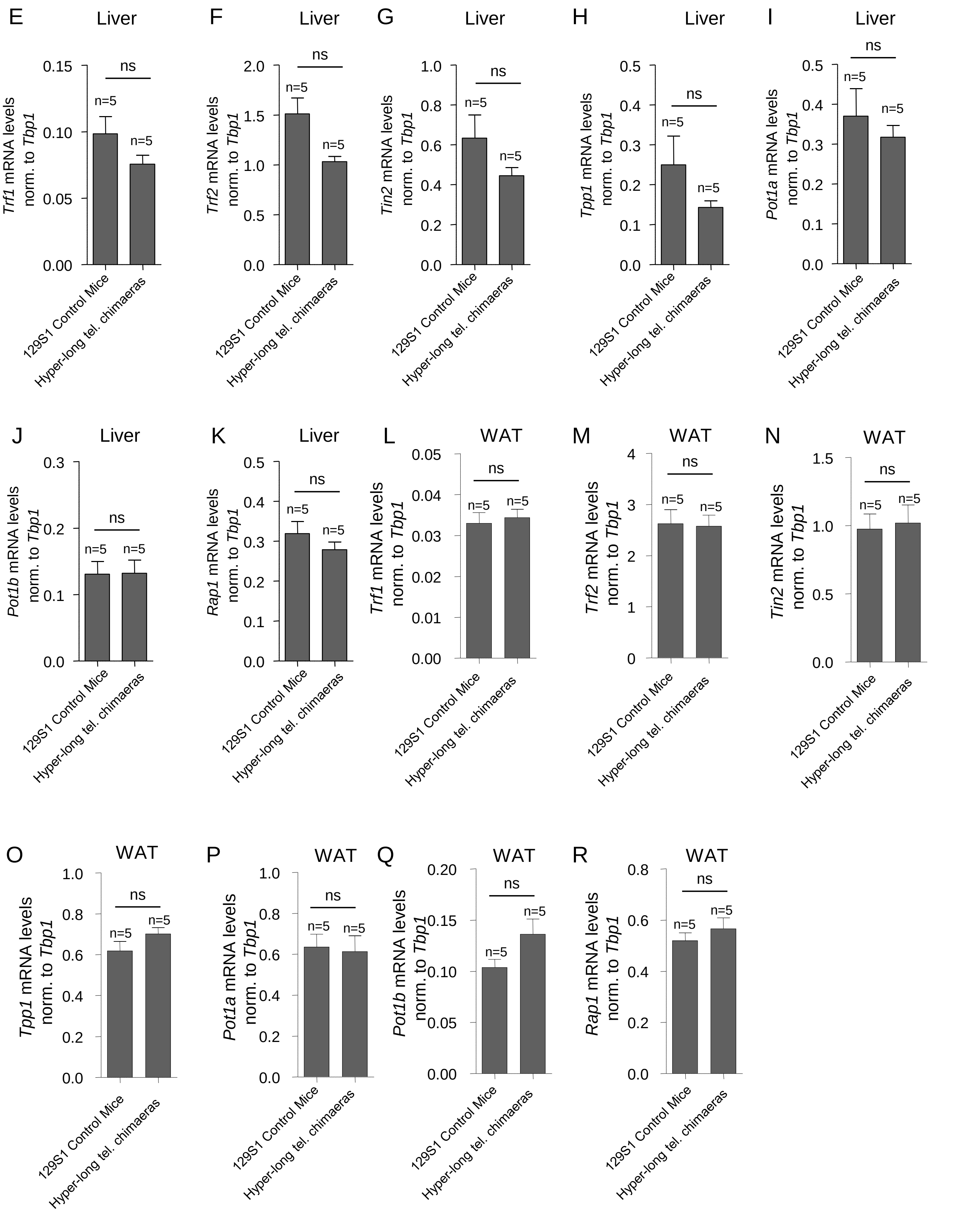

Supplementary Figure 2. Hyper-long telomere mice show normal RAP1 levels. A-B. Western Blot analysis shows RAP1 protein levels in white adipose tissue of age-matched (100 weels old) hyper-long telomere mice and control mice. **C-D.** Immunofluorescence analysis of RAP1 protein levels in the liver of age-matched (100 weeks old) hyper-long telomere mice and control mice. **E-K.** mRNA levels in the liver of shelterins *Trf1* (E), *Trf2* (F), *Tin2* (G), *Tpp1* (H), *Pot1a* (I), *Pot1b* (J) and *Rap1* (K) in 100 weeks-old hyper-long telomere mice and age-matched controls as determined by qPCR. **L-R.** mRNA levels in the WAT of shelterins *Trf1* (L), *Trf2* (M), *Tin2* (N), *Tpp1* (O), *Pot1a* (P), *Pot1b* (Q) and *Rap1* (R) in 100 weeks-old hyper-long telomere mice and age-matched controls measured by qPCR. Error bars represent standard error. *t*-test was used for statistical analysis. The number of mice is indicated in each case.
